# Biomarker-based validation of a food frequency questionnaire for the assessment of omega-3 fatty acid status in a healthy Iranian population

**DOI:** 10.1038/s41598-023-41623-2

**Published:** 2023-09-08

**Authors:** Nasim Abedimanesh, Behrooz Motlagh, Jalal Hejazi, Mohammad Reza Eskandari, Mohammad Asghari-Jafarabadi, Saeideh Mazloomzadeh

**Affiliations:** 1 Social Determinants of Health Research Center, Zanjan University of Medical Sciences, Zanjan, Iran; 2 Department of Clinical Biochemistry, School of Medicine, Zanjan University of Medical Sciences, Zanjan, Iran; 3 Department of Nutrition, School of Medicine, Zanjan University of Medical Sciences, Zanjan, Iran; 4 Department of Pharmacology and Toxicology, School of Pharmacy, Zanjan University of Medical Sciences, Zanjan, Iran; 5 Cabrini Research, Cabrini Health, Malvern, VIC 3144 Australia; 6 School of Public Health and Preventative Medicine, Faculty of Medicine, Nursing and Health Sciences, Monash University, Melbourne, VIC 3800 Australia; 7 Department of Psychiatry, School of Clinical Medicine, Faculty of Medicine, Nursing and Health Sciences, Monash University, Clayton, VIC 3168 Australia; 8 Rajaie Cardiovascular, Medical and Research Center, Tehran, Iran

**Keywords:** Biochemistry, Health care

## Abstract

There is no valid instrument to assess n-3 polyunsaturated fatty acids (n-3 PUFAs) intake in Iran. This study aims to develop a food frequency questionnaire (FFQ) that estimates the intake of n-3 PUFA and validate it in a healthy Iranian population based on the n-3 PUFA content of red blood cells (RBCs) and a 3-day food record (FR). A healthy population (n = 221) was recruited between February and July 2021. Participants completed the new FFQ and 3-day FR to evaluate the average intake of n-3 PUFAs. We used gas chromatography to assess the n-3 PUFA content of RBCs. To validate the FFQ based on FR and biomarker as references, the correlation coefficient was calculated. According to the Bland–Altman plots, a good agreement was found between the new FFQ and FR. Moreover, absolute intake values of ALA, EPA, DPA, DHA, and total n-3 PUFAs based on FFQ were positively correlated to their respective RBC membrane levels (coefficients between 0.205 and 0.508, *p* < 0.005) and FR (coefficients between 0.771 and 0.827, *p* < 0.001). This new FFQ is a valid instrument that can be applied to estimate the n-3 PUFA status of healthy Iranian adults.

## Introduction

The omega-3 (n-3) polyunsaturated fatty acids (PUFAs) exert protective and/or therapeutic effects on numerous diseases associated with inflammation in humans^1^. The n-3 PUFAs, primarily n-3 long-chain PUFAs (n-3 LCPUFAs), such as docosahexaenoic acid (DHA) and eicosapentaenoic acid (EPA), are attributed to a lower risk of cognitive impairment^2^, dementia^3^, depression^4^, cardiovascular disease (CVD)^5^, high blood pressure, and hypertriglyceridemia^6,7^. Alpha-linolenic acid (ALA), as an essential fatty acid, is a precursor of n-3 LCPUFAs. It is a natural source of n-3 fatty acids, mainly found in plant-based oils^8^. ALA can be converted into n-3 LCPUFAs in the human body; however, this conversion is not efficient enough^9,10^. Therefore, supply of EPA, docosapentaenoic acid (DPA) and DHA mainly depends on diet^11^.

Dietary fish and other seafood are rich sources of n-3 LCPUFAs, while meat, eggs, and poultry are minor sources of n-3 LCPUFAs^12^. Fish oil and omega-3 supplements are emerging sources of n-3 LCPUFAs intake, especially for individuals with a low fish intake. To understand the relationship between the n-3 PUFA status and its health consequences and to evaluate the effect of interventions related to n-3 PUFAs, it is important to have an accurate image of their intake, especially in epidemiological studies. There are several methods for the dietary assessment of n-3 PUFAs, including food recalls, food records (FRs), weighed FRs, and food frequency questionnaires (FFQs)^11,13,14^. Of these methods, the FFQ can be useful for several reasons, as it can assess the usual intake and eating habits of an individual or a large population in a limited period at a relatively low cost.

Since n-3 fatty acid-containing foods are limited in the human diet, by designing a simple and short FFQ, the intake of n-3 PUFAs through foods and supplements can be determined almost accurately, without imposing a burden on the respondent especially if we want to assess dietary intake in patients^13,15–17^. However, the new FFQ should include a list of commonly consumed foods by the population. Besides, it is important to assess the validity of this tool in that particular population^15,18^. One of the most extensively used methods for validation of FFQ is evaluating the correlation between the FFQ and two other reference methods, that is, a suitable biomarker and a different dietary assessment instrument^19–22^. The findings of several studies have shown a strong correlation between the results of n-3 PUFA-specific FFQ and health-related biomarkers^4,23,24^. The long-term intake of n-3 PUFAs is reflected in the erythrocyte membrane dose-dependently. Therefore, erythrocytes are considered a suitable biomarker of n-3 PUFA intake^25^. So far, numerous FFQs have been designed and validated to measure the n-3 PUFA intake in various populations^4,23,24,26^.

Based on our literature review, there is no valid Persian version of FFQ to evaluate the n-3 PUFA status. Therefore, the present study aimed to develop a specific FFQ to investigate the n-3 PUFA intake of an Iranian population and to validate it by using two other reference methods, including a 3-day FR and red blood cell (RBC) n-3 fatty acid composition.

## Results

The characteristics of study population are introduced in Table 1. Participants were between 18 to 45 years old. The number of men and women was almost the same. Over 60% of participants were school graduated and only 18% of the participants were smokers. Thirty-six percent (36%) of participants received n-3 PUFA or fish oil supplements several days during every month.

**Table 1:** Characteristics of the study population.

	Total participants (n = 200)	
	Mean or frequency	SD or percentage
Age (years)	29.94	7.82
BMI (kg/m^2^)	27.09	2.63
Sex		
Men (%)	104	52
Women (%)	96	48
Education		
High school (%)	123	61.5
University educated (%)	77	38.5
Smoking		
Yes (%)	36	18
No (%)	164	82

Data are presented in mean (SD) or frequency (percentage).

*BMI* body mass index.

### Validation of FFQ based on FR

**Figure 1:**
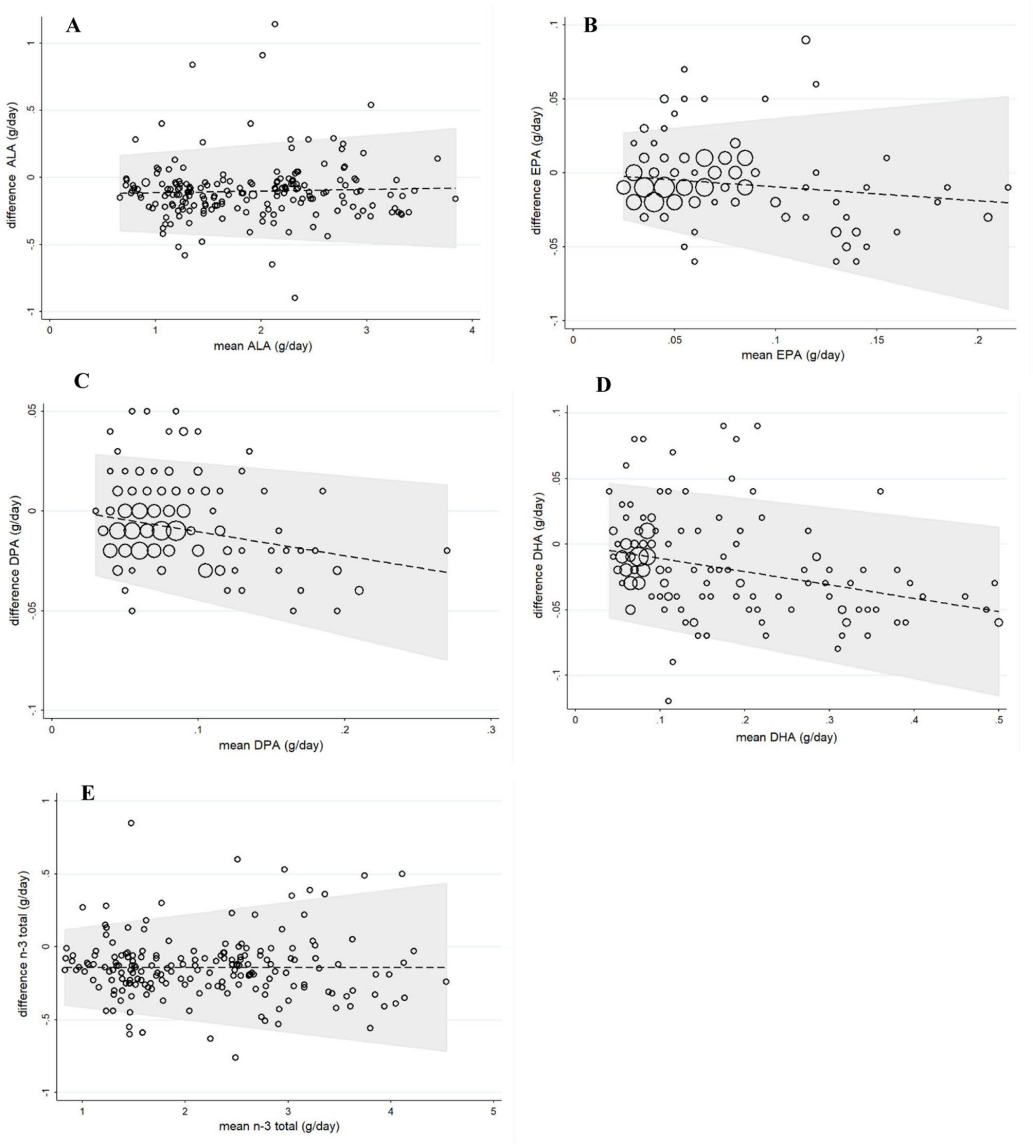
Bland Altman plots showing the agreement between 3-day food records and food frequency questionnaire to assess the intake of: (**A**) α-linolenic acid (ALA), (**B**) eicosapentanoic acid (EPA), (**C**) docosapentanoic acid (DPA), (**D**) docosahexanoic acid (DHA), and (**E**) total n-3 polyunsaturated fatty acid intake (total n-3).

Mean energy intake of participants was 2491.51 ± 351.99 kcal (Median: 2547.75, 25th–75th: 2176.22–2781.92) based on food records. The mean percent of carbohydrate intake was 53.82 ± 2.96 (Median: 53.65, 25th–75th: 51.62–55.80), protein intake was 16.64 ± 1.35 (Median: 16.70, 25th–75th: 15.70–17.40) and fat intake was 29.54 ± 3.09 (Median: 29.75, 25th–75th: 27.50–32.00). Daily dietary intake of n-3 PUFA values based on 3-day FR and Persian FFQ are shown in Table 2. Also in this table, the results of correlation analysis of the values obtained from FFQ and FR are demonstrated. The significant correlations with a Spearman correlation coefficient between 0.771 and 0.827 between FR and FFQ was determined (*p* < 0.001). The findings of the agreement between FFQ and FR for ALA, EPA, DPA, DHA and total n-3 PUFA are depicted in the form of the Bland Altman plots (Fig. 1). The results of quantile regression were non-significant for ALA (β = -0.009, *p* = 0.648), EPA (β = 0.085, *p* = 0.053), DPA (β = 0.076, *p* = 0.062), total n-3 PUFA (β = 0.002, *p* = 0.896), indicating no proportional bias. According to the results of Wilcoxon signed rank test, daily intake of n-3 PUFAs based on new FFQ was significantly higher than 3-day FR (*p* < 0.001). However, the percentage change in the values obtained from the FFQ and the FR was not significant (less than 5%).

**Table 2:** Daily dietary intake of n-3 PUFA in study population assessed using 3-day food records and FFQ and correlations of n-3 PUFA intakes between FR and FFQ (n = 200).

	Food records (g/day)			FFQ (g/day)			Correlations	
	Median	Interquartile range		Median	Interquartile range		Spearman’s r	*p*-value^a^
		P25	P75		P25	P75		
ALA	1.77	1.17	2.40	1.78	1.28	2.44	0.826	< 0.001
EPA	0.06	0.03	0.08	0.06	0.04	0.08	0.776	< 0.001
DPA	0.07	0.05	0.09	0.08	0.06	0.09	0.771	< 0.001
DHA	0.09	0.07	0.26	0.09	0.08	0.21	0.798	< 0.001
Total n-3 PUFA	2.06	1.37	2.78	2.15	1.54	2.84	0.827	< 0.001

Data are presented in median, percentiles 25 and 75.

*ALA* α-linolenic acid, *EPA* eicosapentanoic acid, *DPA* docosapentanoic acid, *DHA* docosahexanoic acid.

^a^Based on Spearman’s correlation test.

### Validation of FFQ based on RBC n-3 PUFA content

Dietary intake of n-3 PUFA assessed by the Persian n-3 PUFA FFQ, RBC’s membrane n-3 PUFA percentages, and the results of the correlation and regression analyses are shown in Table 3. We observed strong to moderate correlation with DHA (r = 0.410) and total n-3 PUFA (r = 0.508) and weaker correlation with DPA (r = 0.205) and EPA (r = 0.268). Based on the quantile regression findings after adjusting for control variables such as gender, supplement intake (yes, no) and marine sources of n-3 PUFA intake (low, medium and high intake), daily intakes of n-3 PUFAs according to FFQ were significantly correlated with RBC n-3 PUFA percentages.

**Table 3:** Dietary intake of n-3 PUFA in study population assessed by FFQ and percentage of n-3 fatty acid content of the erythrocyte membrane, as well as correlations and regressions between the two (n = 184).

	FFQ (g/day)			% RBC membrane composition			Correlations		Regression	
	Median	Interquartile range		Median	Interquartile range		Spearman’s r	*p*-value^a^	B (SE)	*p*-value^b^
		P25	P75		P25	P75				
ALA	1.81	1.32	2.45	0.09	0.07	0.14	0.366	< 0.001	0.02 (0.01)	< 0.001
EPA	0.06	0.04	0.08	1.12	0.99	1.22	0.268	< 0.001	1.36 (0.33)	< 0.001
DPA	0.08	0.06	0.09	2.06	1.96	2.22	0.205	0.004	1.32 (0.40)	0.001
DHA	0.09	0.08	0.17	4.86	4.67	5.29	0.410	< 0.001	1.98 (0.36)	< 0.001
Total n-3 PUFA	2.18	1.55	2.85	8.10	7.73	8.83	0.508	< 0.001	0.55 (0.06)	< 0.001

Data are presented in median, percentiles 25 and 75.

*ALA* α-linolenic acid, *EPA* eicosapentanoic acid, *DPA* docosapentanoic acid, *DHA* docosahexanoic acid.

^a^Based on Spearman’s correlation test.

^b^Based on quantile regression adjusted for control variables (gender, supplement intake, marine sources of omega-3).

## Discussion

The present study aimed to determine whether the adapted Persian version of the FFQ, which was completed by a healthy Iranian adult population, could adequately estimate the dietary intake of n-3 PUFAs, using a 3-day FR and quantification of RBC n-3 PUFAs as references. The new FFQ was adapted from the validated n-3 PUFA-specific FFQ used by Sublette et al. and then localized for the Iranian dietary culture^4^. Based on our literature review, there is no valid or specific n-3 PUFA FFQ for the Iranian population. Therefore, the n-3 PUFA status was assessed in individuals using a non-specific FFQ with more food items and time. The results of this study demonstrated that our specific, simple, and short FFQ could provide adequate and accurate information on the n-3 PUFA intake of the participants. The validity of this new FFQ was evaluated and confirmed based on the fatty acid composition in the erythrocyte membrane and the 3-day FR.

The present findings showed that the daily intake levels of n-3 PUFAs, based on the new FFQ and FR, were significantly correlated; the correlation coefficients were between 0.771 and 0.827. Consistent with our findings, in several previous studies, a significant correlation was observed between the FFQ and FR results, although the correlation coefficients were comparable^13,19,27^. The time period covered by the FFQs differed among these validation studies, which assessed the dietary intake of healthy adults in the past 3 months, in the last year, and in the past 6 months^13,19,27^. The current study investigated the dietary intake of the participants in the past 6 months. All previously mentioned studies used the 3-day FR method, except for the study by Herter-Aeberli et al.^13^, which used the seven-day FR^19,27^; these factors may explain the difference between the correlation coefficients of FFQ and FR in these studies.

The high correlation coefficients measured in the present study may be related to the fact that the questions of the new FFQ fully covered common n-3 PUFA-rich food sources in the study population; also, the questions were simple with an easy estimation of portions and a short interval between the completion of FFQ and the three-day FR. Besides, the new FFQ estimated higher intakes of ALA, EPA, DPA, DHA, and total n-3 PUFAs compared to the FR method in this study; however, the percentage difference between the two methods was below 5% and insignificant. In similar studies, the FFQ estimated higher intakes of DPA, EPA, DHA, and ALA (or some of them) compared to the FR^13,19,20,27,28^.

Almost all n-3 PUFA-rich foods, including fish and seafood as rich sources of EPA and DHA, meat as a source of DPA, and some plant-based foods (e.g., walnut and flaxseed) and some oils (flaxseed and canola oils) as sources of ALA were included in our new FFQ. Since the number of n-3 PUFA-fortified foods is very limited in Iran, they are not generally considered common dietary sources of n-3 PUFAs for people. Differences in n-3 PUFAs between the FFQ and FR might have contributed to day-to-day intake variabilities. For example, some participants might have consumed n-3 PUFA-rich foods several times in the last 6 months, which was not reported in the three-day FR. Another explanation for this finding is that people usually underreport their food intake when completing the FR. Similar reasons have been suggested by researchers in several similar studies^13,19,27^.

It is known that meat is a good source of n-3 PUFAs, especially DPA (22:5, n-3)^28^. However, some studies developing an n-3 PUFA-specific FFQ did not include meat in the questionnaire^4,13^. Since the consumption of lamb, beef, veal, and poultry is more common than fish and seafood in the Iranian population (especially in populations living farther from the northern and southern parts of the country facing the sea), these items were included in the new FFQ. To compare the results of FFQ and FR, a Bland–Altman agreement analysis was performed; the plots indicated differences between the methods based on the mean values of these two tools. According to the findings of this study, there was an acceptable agreement between the results of these two methods, which is in line with the results of other studies^13,27^. Generally, the closer the mean difference is to zero, the higher the agreement is. Also, more points within the limits of agreement confirmed the validity of the new FFQ.

In the present study, the n-3 PUFA content of RBCs was comparable to that of previous studies, and almost high values of ALA, EPA, DPA, DHA, and total n-3 PUFAs were reported^13,29^. This finding can be explained by storage conditions of samples and the duration. In this population the incorporation of n-3 PUFAs may be higher and can be either explained by dietary intake or higher metabolism rates amongst other. Genetic factors may also play a role. The longer this process is, or the longer the RBCs are stored, the more likely n-3 PUFAs are to degrade^29,30^. In the present study, the mean levels of ALA (0.10), EPA (1.11), DPA (2.10), DHA (4.99), and total n-3 PUFAs (8.31) were estimated as the percentage of total fatty acids in RBCs, which is rather similar to the results of a study by Sun et al.^31^.

The level of erythrocyte phospholipids is the standard and most frequently used biomarker for evaluating the long-term status of n-3 PUFAs^25,32^. This biomarker was used to assess the validity of the new FFQ in our study. The current findings showed significant correlations between the n-3 PUFA-specific FFQ results and RBC n-3 PUFA composition, suggesting the potential use of the newly developed FFQ for the assessment of dietary ALA, EPA, DPA, DHA, and total n-3 PUFAs. It is important to note that after adjusting for some control variables, such as sex (m/f), supplement intake (yes/no), and marine-derived sources of n-3 PUFAs (low, moderate, and high intake), the results of the quantile regression were found to be significant, as well.

Consistent with our findings, a significant correlation was found between the EPA and DHA contents of RBCs or blood samples based on the n-3 PUFA-specific FFQ in the general population of several studies^19,23,32^. However, the correlations were less significant than the correlations between the FFQ and FR results in the present study. Consistent with the current study, Swierk et al.^19^ and Sullivan et al.^23^ found a significant correlation between the total n-3 PUFA content of the erythrocyte membrane and the FFQ data on the n-3 PUFA-specific intake. Compared to the mentioned studies, our new FFQ showed higher Spearman’s correlation coefficients for both DHA and total n-3 PUFAs^19,23^. The increased validity coefficient may be related to the specific and well-designed PUFA FFQ for evaluating n-3 PUFAs, as well as the very short interval between the FFQ completion and the erythrocyte membrane analysis.

In line with the findings reported by Swierk et al.^19^ and Parker et al.^33^, a significant correlation was found between the erythrocyte membrane DPA content and the FFQ results. On the other hand, Sullivan et al.^23^ reported inconsistent results. DPA incorporates differently to the phospholipids of plasma and/or erythrocytes, which may explain the controversial results^34^. Generally, ALA is usually not well incorporated into cell membranes although it is an essential fatty acid and fully dependent on dietary intake. It tends to be distributed in different tissues in large or small amounts. It can be metabolized in different ways, including desaturation and elongation to synthesize n-3 LCPUFAs. Consequently, the concentration of ALA in the cell membrane is often low and may not be a proper representative of the n-3 status.

In the present study, there was a moderate correlation (r = 0.366) between the FFQ and RBC n-3 ALA content, which is consistent with the results of other studies^13,29^. However, in several studies, no significant correlation was observed between the dietary intake of ALA and biomarkers. The metabolism of ALA and its conversion to LC-PUFAs are influenced by n-3 levels, as well as different genetic variants of key enzymes, including fatty acid desaturase 1 (FADS 1) and FADS 2^35^. In other words, the polymorphisms of FADS1 and FADS2 are not the same in different populations, which probably explains the contradictory findings of various studies.

One of the limitations of the present study is that the short FFQ cannot estimate energy and fat intake accurately; therefore, it was not possible to adjust for these factors. Also, the reproducibility of the FFQ was not investigated. Using a 3-day versus 7-day FR is another limitation of study. The validity of the FFQ was only evaluated in a healthy population and cannot be generalized to patients. Also it should be noted that this study was conducted on a population who mostly had an academic education (more than 60%) and probably had higher levels of nutritional knowledge and information compared to the general population. Economic status is also one of the factors that can affect the n-3 PUFA intake. Due to the high cost of n-3 PUFA-rich food sources in Iran, it is not possible to provide these items to everyone equally. Therefore, the level of literacy and income of the study population can be confounding factors and may be considered in future studies.

The present findings revealed that n-3 PUFA dietary intake, which was calculated using the new n-3 PUFA FFQ, was correlated with the standard biomarker of n-3 PUFA status. In conclusion, the new FFQ can be considered a valid questionnaire to specifically evaluate the n-3 PUFA status in the Iranian population.

## Materials and methods

### Study population and design

**Figure 2:**
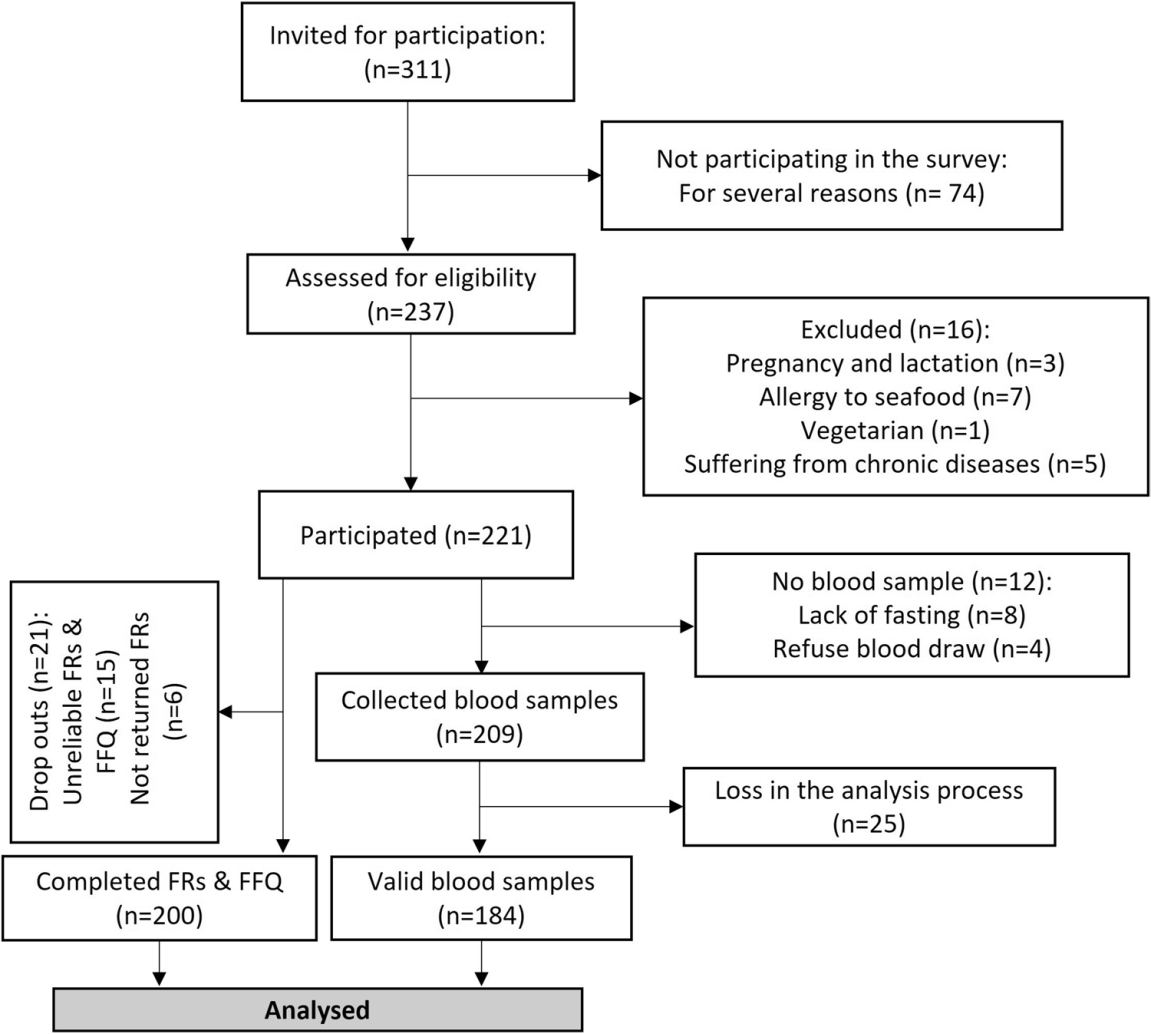
Flow diagram of the study population selection.

A total of 221 healthy individuals participated in this cross-sectional study. The final sample included 200 participants (104 men and 96 women), aged 18–45 years. The study flow diagram is illustrated in Fig. 2. Poster advertisements were used to randomly recruit volunteers from the general population between February and July 2021. The inclusion criteria were as follows: not being a vegetarian; not being allergic to seafood; lack of chronic diseases or major depression according to the participant’s self-report; not being pregnant or lactating; and adherence to a consistent diet in the past three months. Written informed consent was obtained from all subjects and/or their legal guardian(s). This study was approved by the Zanjan University of Medical Sciences Ethics Committee (IR.REC.ZUMS.1397.253) and all methods were performed in accordance with the relevant guidelines and regulations.

### Dietary intake assessment of n-3 PUFAs

#### FFQ

The FFQ used in this study was designed and developed based on a sample n-3 PUFA-specific FFQ by Sublette et al.^4^. All food items in the sample FFQ were included in our new FFQ. Several food items were also added to the FFQ. These two FFQs were different regarding the duration of food intake assessment and also the portion sizes. The new FFQ was a short, feasible, and self-report questionnaire, which consisted of 14 questions on 12 food items, mainly consisting of n-3 PUFAs in the Iranian food culture, as well as two other questions on supplements; eight out of these 12 food items were similar to the sample FFQ. Moreover, given the relatively high consumption of red meat, poultry, and processed meat as major sources of n-3 PUFAs among Iranians, these three food items were included in the FFQ. Although the consumption of omega-3-fortified eggs is not very common among Iranians, it was added to the FFQ list. The portion sizes of food items were defined based on the most commonly consumed portion sizes for each item in the Iranian general population.

**Figure 3:**
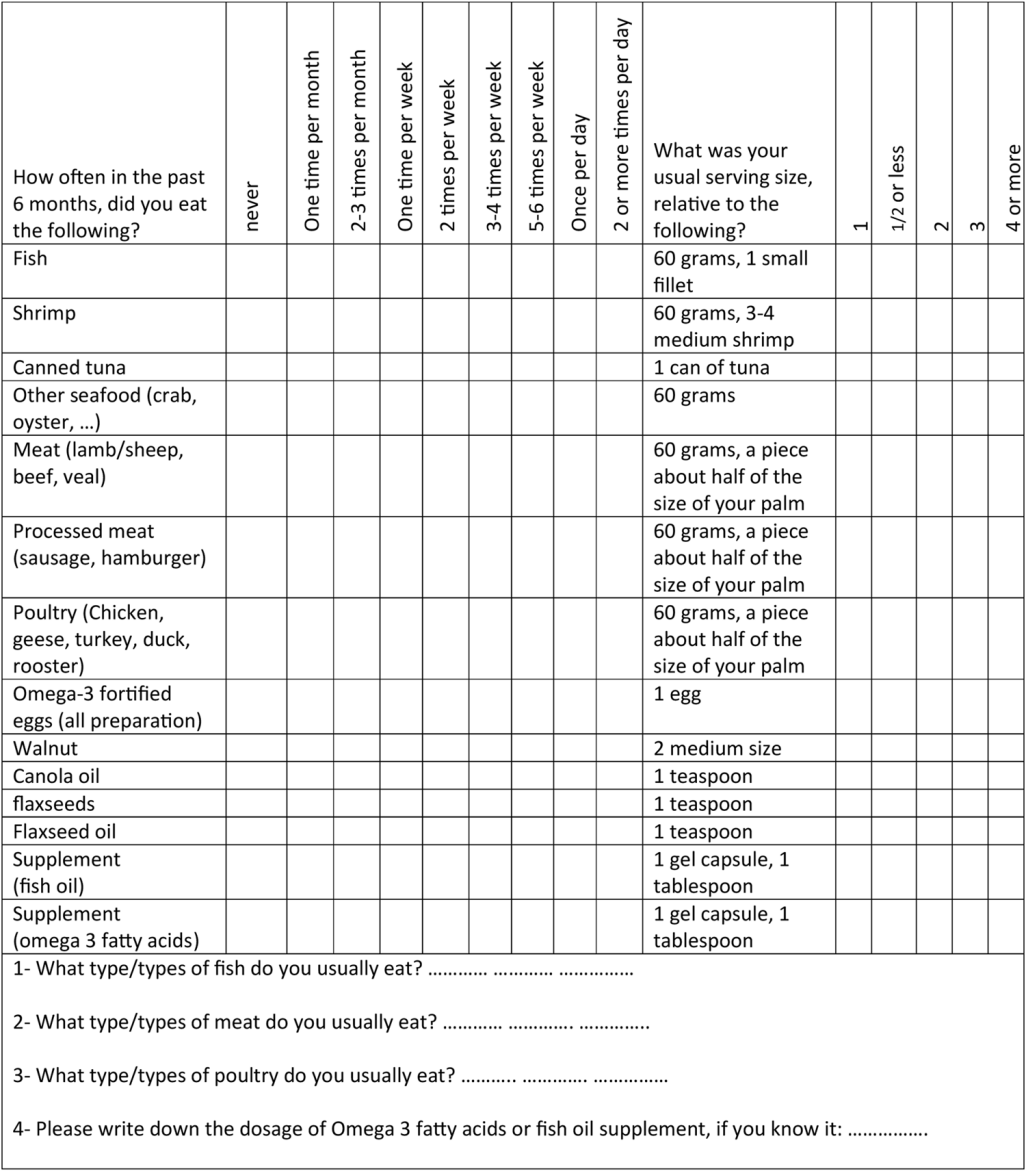
Questions asked on new FFQ.

To familiarize the participants with the portion sizes and the frequency of consumption and also to select the most suitable option from the FFQ list and reduce possible errors, a 30-min training session was held by a skilled nutritionist at the study center. In this session, the portion sizes were illustrated with pictures or using household scales. The participants’ daily, weekly, and monthly consumption of fish, shrimp, and other seafood, as well as all types of meat (i.e., red, processed, and white meat), was expressed in grams; the consumption of canned tuna, omega-3-fortified eggs, and walnuts was expressed in number; the consumption of canola cooking oil, flaxseed, and flaxseed oil was expressed as the number of teaspoons; and the consumption of omega-3 and/or cod liver oil capsules were expressed in number (in tablespoons if liquid) in the new FFQ. Besides, the type of typically consumed fish and meat was examined. The new FFQ is presented in Fig. 3.

#### FR method

The estimated FR method provides food intake information, including the type of food, the consumed amount, preparation methods, ingredients, and used oils during 24 h for 3 non-consecutive days. This method was also used in the present study to evaluate the mean daily intake of calories, macronutrients, total n-3 PUFAs, and its components.

### Analysis of FFQ and FR methods

The nutritional information of FFQ and FR was entered in Microsoft Excel software by a skilled nutritionist. The average daily intake of food items was determined and then processed using the Nutritionist IV software, modified for Iranian food items (Hearst Corporation, San Bruno, CA, USA). The daily dietary intake of n-3 PUFAs was reported based on the new FFQ and FR for all participants (in gram per day).

### Study procedure

After the study goals and procedure were explained to interested volunteers, their demographic and anthropometric information (i.e., weight, height, and body mass index) was recorded by a research team. The participants were trained on how to complete the FFQ and FR by a skilled nutritionist. Since some food items and portion sizes in the FFQ could be unfamiliar to the participants, each food item was introduced with pictures or real samples. The portion sizes of meat, fish, walnut, and shrimp were illustrated in a food album. Samples of teaspoon and tablespoon were also shown to the participants.

To complete the FRs, the participants were asked to report the type of consumed food, the amount of consumed food in usual household scales (e.g., plates, bowls, glasses, and spoons), preparation methods (e.g., boiling, steaming, grilling, and frying), the amount and type of oils used, and the amount and type of ingredients in mixed foods if consumed. Next, they were asked to attend the central laboratory of the medical school following overnight fasting to provide a blood sample for the analysis of RBC fatty acids and to complete the FFQ. Three copies of FR sheets were presented to the participants to complete at home within the next 10 days (2 week days and a weekend day), without changing their eating habits, and to return them to the researcher.

### Preparation and analysis of erythrocyte membrane fatty acid composition

The methodology for the assessment of fatty acids and the chromatographic method was based on Sullivan et al. and Ridges et al.^23,36^. Fasting blood samples (5 mL) were collected via venipuncture into the tubes containing EDTA. Erythrocytes were separated from fresh whole blood by centrifugation at 1000×*g* at 4 °C for 10 min. To remove buffy coats, erythrocytes were washed with ice-cold phosphate buffer saline and centrifuged at 1000×*g* for 10 min at 4 °C. The precipitated erythrocytes were lysed with cold distilled water. For trans-esterification of fatty acids, the mixture of methanol: toluene (4:1) was added, and while vortexing, acetyl chloride was also added dropwise. Samples heated at 100 °C for 1 h and then cooled in a cold water. To extract the fatty acid methyl esters, potassium chloride (10%) and toluene were added to each sample. After centrifugation, the supernatant containing methyl ester fatty acids in toluene is collected into GC vials and stored in − 80 °C for further gas chromatography analysis. For this, a flame-ionization gas chromatography (Agilent 6890N instrument) with a 30 m × 0.25 mm internal diameter capillary column (Agilent; 122-0132UI) was used. Helium and nitrogen were used as carrier and make-up gases respectively. The split ratio was 17: l, and the injection port and detector temperature were 200 and 250 °C respectively. The column temperature was set to 80 °C for 5 min and in a step-wise manner reached to a plateau of 220 °C. Individual fatty acids were identified in a proportional manner to known fatty acid standards (18919-1AMP, FAME Mix, C4-C24, Sigma-Aldrich). Out of 188 blood samples taken, the results of RBC n-3 PUFA content were reported for 184 valid samples. Medians and interquartile ranges were reported for the total n-3 PUFA, ALA, EPA, DPA and DHA content (as a percentage of total fatty acids) of the RBCs.

### Sample size justifications

The sample size of the study was estimated considering the minimum correlations between main outcomes of the study. Allowing for a correlation of 0.2, a power of 0.8, a confidence level of 0.95, the minimum sample size required for the study was at least 195, which increased to 200 for slight increase in the precision.

### Statistical analysis

The normality of data was evaluated by skewness within ± 1.5 and kurtosis within ± 2. Non-parametric tests were conducted when the variables did not show a normal distribution. Data were expressed using mean (SD) and median (percentile25–percentile75) for normal and non-normal numeric variables, respectively, and frequency (percent) for categorical variables. The relationship among the new FFQ, 3-day FR, and the biomarker was assessed. Wilcoxon signed rank test was carried out to compare between new FFQ and 3-day FR in daily intake of n-3 PUFAs. Moreover, the direction and strength of the association between the 3-day FR and FFQ were assessed using Spearman’s correlation coefficients. To assess the agreement between the two measurement tools for ALA, DPA, EPA, DHA and total n-3 PUFA intake, Bland Altman Plots were carried out. The differences of the two measurements were plotted against the mean of the two methods. To assess the relationship between dietary intake of n-3 PUFA by FFQ and percentage of n-3 fatty acid content of the erythrocyte membrane, the Spearman’s correlation coefficients were used and to investigate the effect of control variables such as gender, supplement intake and marine sources of n-3 PUFA, quantile regression was conducted. STATA (version 16, Stata Corp, College Station, Texas USA) was used to conduct analyses. *p*-values < 0.05 considered as significant.

## Data Availability

All data generated or analyzed during this study are included in this published article.
